# Differences in the Synovial Fluid Proteome of Septic and Aseptic Implant Failure

**DOI:** 10.3390/antibiotics13040346

**Published:** 2024-04-09

**Authors:** Andrea Sowislok, André Busch, Farnusch Kaschani, Markus Kaiser, Marcus Jäger

**Affiliations:** 1 Chair of Orthopedics and Trauma Surgery, University of Duisburg-Essen, 45147 Essen, Germany; andrea.sowislok@uni-due.de; 2 Department of Orthopedics, Trauma and Reconstructive Surgery, Katholisches Klinikum Essen Philippus, 45355 Essen, Germany; andre.busch@sana.de; 3 Analytics Core Facility Essen (ACE), ZMB, Chemical Biology, University of Duisburg-Essen, 45141 Essen, Germany; farnusch.kaschani@uni-due.de; 4 Chemical Biology, Faculty of Biology, University of Duisburg-Essen, 45141 Essen, Germany; markus.kaiser@uni-due.de; 5 Department of Orthopedics, Trauma and Reconstructive Surgery, St. Marien Hospital Mülheim a. d. Ruhr, 45468 Mülheim, Germany

**Keywords:** periprosthetic joint infections, synovial fluid, proteome, biomarker, *LRG1*

## Abstract

Implant loosening is a severe complication after total joint replacement. Here, differential diagnosis between septic and aseptic cases is crucial for further surgical treatment, but low-grade periprosthetic joint infections (PJIs) in particular remain a challenge. In this study, we analyzed the synovial fluid proteome of 21 patients undergoing revision surgery for septic (eight cases) or aseptic (thirteen cases) implant failure using LC-MS/MS to identify potential new biomarkers as future diagnostic tools. *Staphylococci* were found in four cases, *Streptococci* in two cases, *Serratia marcescens* and *Cutibacterium acnes* in one case. Proteomic analysis of the synovial fluid resulted in the identification of 515 different proteins based on at least two peptides. A statistical comparison revealed 37 differentially abundant proteins (*p* < 0.05), of which 17 proteins (46%) showed a higher abundance in the septic group. The proteins with the highest fold change included the known marker proteins c-reactive protein (7.57-fold) and the calprotectin components protein *S100-A8* (4.41-fold) and protein S100-A9 (3.1-fold). However, the protein with the highest fold change was leucine-rich alpha-2-glycoprotein 1 (*LRG1*) (9.07-fold), a currently discussed new biomarker for inflammatory diseases. Elevated *LRG1* levels could facilitate the diagnosis of PJI in the future, but their significance needs to be further investigated.

## 1. Introduction

In the Western world, total joint replacement is a highly standardized procedure that improves mobility and quality of life, especially in the elderly population. In Germany, more than 300,000 patients underwent either total hip (177,826 patients) or total knee arthroplasty (137,030 patients) in 2022 [1]. Due to the aging population, high obesity rates, and the desire to remain physically active in old age, the number of annual joint replacement operations is expected to increase even further in the future. For this reason, the failure of endoprostheses will also increase, including the burden on patients and higher costs for the healthcare system [2]. Today, aseptic implant loosening is a serious complication after total joint replacement and accounts for more than 22% of all revision procedures followed by periprosthetic joint infections (PJIs), which account for 16% [1]. Aseptic implant failure includes periprosthetic fracture, mechanical instability, osteolysis due to wear debris, and stress shielding. Early PJIs, occurring less than three months after initial implantation, show typical signs of infection and are mainly caused by germs of high virulence, such as methicillin-resistant staphylococci, enterococci, or Gram-negative bacilli. Delayed (3–24 months after implantation) or low-grade PJIs are caused by low-virulence organisms such as methicillin-sensitive and coagulase-negative *Staphylococci* (CoNS), *Streptococci* and *Propionibacterium acnes* [3]. These bacilli cause the formation of resistant biofilms on the implant and the surrounding tissue by producing immunomodulating products and extracellular polymeric substances, leading to resistance to antibiotics and the host’s immune response as well as to a reduced possibility of microbial detection [4]. In surgical treatment, the differential diagnosis between septic and aseptic cases is crucial, as septic implant failures require treatment with antimicrobial agents and sometimes a two-stage revision, including implant removal, debridement, and the implantation of an antibiotic-releasing spacer prior to re-implantation [5]. However, rapid and accurate clinical differentiation between septic and aseptic implant failure remains a challenge, especially in cases of low-grade PJI, where the usual clinical signs of infection may be absent. Diagnostic tests and clinical features may be inconsistent and to date, there is no diagnostic “gold standard” [6]. In general, the diagnosis of PJI is based on a combination of clinical and intraoperative findings, microbiologic culture, histologic evaluation of periprosthetic tissue, and laboratory testing of blood and synovial fluid. In 2011, the Musculoskeletal Infection Society (MSIS) working group proposed a set of major and minor criteria for the definition of a PJI to address the lack of a single recognized set of diagnostic criteria for a PJI and the often inconsistent definitions [7]. In the following years, at least five different definitions of PJI were proposed [8,9,10,11,12]. According to the 2018 consensus definition for PJI, the major criteria for the diagnosis of PJI are the presence of a sinus tract with evidence of joint communication or visualization of the implant or at least two positive cultures of periprosthetic tissue. Minor criteria include synovial fluid biomarkers such as c-reactive protein (CRP), α-defensin, leucocyte esterase, and synovial blood cell composition such as white blood cell count (WBC) or polymorphonuclear leucocyte percentage (PMN%) [12]. However, the accuracy of these markers is limited, and it has been shown that choosing the right marker is not easy due to its advantages and disadvantages [13]. Microbiological diagnosis occasionally produces a false negative or positive [14]. It has been shown that the results of the WBC count and PMN values in the synovial fluid are distorted if antibiotics are administered before joint aspiration, if the synovial fluid is mixed with blood, or if there is open pus or inflammatory arthritis. Serum erythrocyte sedimentation rate (ESR) and serum CRP can be affected by various systemic inflammatory conditions such as autoimmune disorders, obesity, non-joint-related infections, and others. In contrast, alpha-defensin determination remains accurate even in the presence of antibiotics and blood admixtures in the synovial fluid, but is costly and has limited accessibility, and studies have shown that it is not statistically superior to the combination of the WBC count and the PMN% in the synovial fluid [13,15]. Research into new reliable biomarkers for the diagnosis of PJI is ongoing, focusing on some new markers such as procalcitonin [16], calprotectin [17], IL-1β [18], and other immune system-related proteins. However, these markers need stronger evidence in order to be used as diagnostic markers [19,20,21,22]. 

In this study, we used liquid chromatography-coupled mass spectrometry (LC-MS/MS) to compare the protein composition of the synovial fluid from patients scheduled for revision arthroplasty due to septic or aseptic implant failure to find potential synovial biomarkers that could serve as possible diagnostic tools in the future.

## 2. Results

### 2.1. Patient Groups and Characteristics

According to the definition of Parvizi et al. [12], thirteen patients (62%) were assigned to the aseptic group and eight patients (38%) to the PJI group. The aseptic group consisted of two men and eleven women with a mean age of 67.8 ± 11.0 (52–85) years at the time of surgery, and revision procedures were performed on seven hips and six knees (Table 1).

Causes of implant failure and indication for revision surgery included aseptic loosening of the implant in nine cases (69%), instability in two cases (15%), and periprosthetic fracture and restriction of movement, each in one case (7.5%). The PJI group consisted of four men and four women, with a mean age of 68.5 ± 13.3 (51–90) years at the time of surgery, and revision procedures were performed on six hips and two shoulders (Table 1). Microbiological examination of the tissue samples taken intraoperatively revealed *Staphylococci* in four cases (50%), *Streptococci* in two cases (25%), *Serratia marcescens* (12.5%) and *Cutibacterium acnes* (12.5%) in one case. There were no significant differences in sex, age, and BMI between the cohorts (*p* > 0.05, Table 2). With the exception of CRP and creatinine, the preoperative blood parameters of the patients were comparable in both groups, were within the normal range, and showed no statistical significance (*p* > 0.05, Table 1). Creatinine levels were slightly higher in the septic group, without this being statistically significant (*p* = 0.514). Serum CRP levels were significantly higher in the septic group (*p* = 0.002) at 5.1 mg/dL (0.4–17.2) compared to 0.5 mg/dL (0–2.5) in the aseptic group.

### 2.2. Proteome Analysis of the Synovial Fluid

#### 2.2.1. General Composition of the Proteome

**Figure 1:**
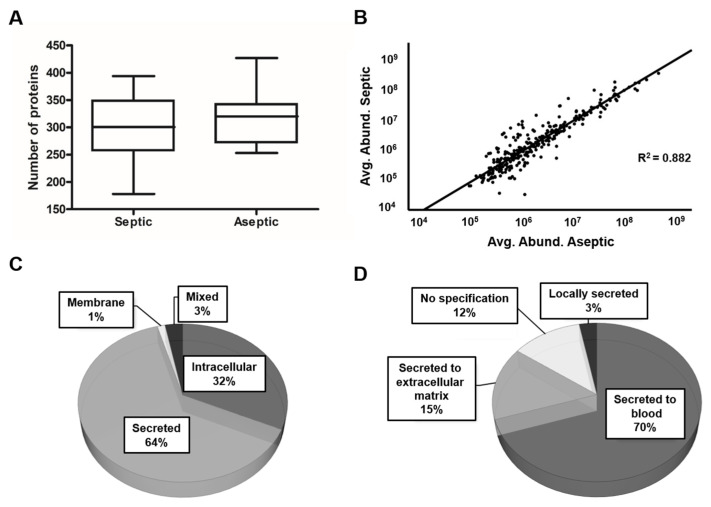
Overview of the SF proteomics data. (**A**): The box plot shows the distribution of the number of identified proteins (≥2 peptides) per sample over the analyzed conditions. (**B**): The scatter plot with linear regression shows the relationship between the average values of the relative abundance of each of the identified proteins in the SF of each condition. (**C**): A pie chart shows the subcellular localization of the proteins based on the data from the Human Protein Atlas. (**D**): Further specification of the localization of the secretion.

To compare the proteomic profile of the synovial fluid of the patients in each group, LC-MS/MS-based proteomics was performed. Proteomic analysis of the SF resulted in the identification of 515 different proteins based on at least two peptides. To investigate proteomic differences between septic and aseptic implant failure, the proteomic profiles were separated into the two groups: septic (n = 8) and aseptic (n = 13). The average number of proteins identified in septic samples was 298 ± 69 (178–394) and 316 ± 50 (253–427) in aseptic samples, with no statistical differences (*p* = 0.492, Figure 1A).

To further increase the confidence level of the proteomic data and for the subsequent statistical analysis, only proteins that occurred in 60% of all samples within one group were considered, resulting in a protein count of 305. Of these proteins, 64% (194) were classified as secreted proteins, 32% (97) were of intracellular origin, 1% (4) were assigned to the membrane fraction, and 3% (10) could be assigned to more than one category, depending on the isoform (Figure 1C). Among the secreted proteins, 70% (135) were secreted into the blood, followed by 15% (30) that were secreted into the extracellular matrix (Figure 1D). Linear regression revealed a strong positive and significant relationship between the mean values of the relative abundance of the identified proteins in the septic and aseptic group (Figure 1B, F (1303) = 2260.012, *p* < 0.001), indicating a good consistency of the results.

**Figure 2:**
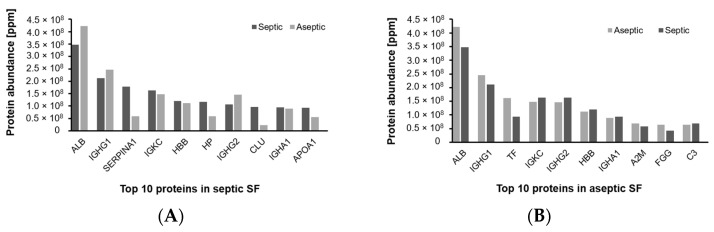
Differential comparison of the composition of the SF proteome within the two groups. (**A**): Ranking of the top 10 most abundant proteins in the SF of patients with septic implant failure in comparison to aseptic implant failure. (**B**): Ranking of the top 10 most abundant proteins in the SF of patients with aseptic implant failure in comparison to septic implant failure.

Depending on the reference set used, 38–87% of the proteins could be assigned to the plasma proteome. A closer look at the ten proteins with the highest abundance showed that serum albumin (*ALB*) and immunoglobulin heavy constant gamma 1 (*IGHG1*) were found in the first two positions in SF of patients with both septic and aseptic implant failure (Figure 2).

#### 2.2.2. Differentially Abundant Proteins

**Figure 3:**
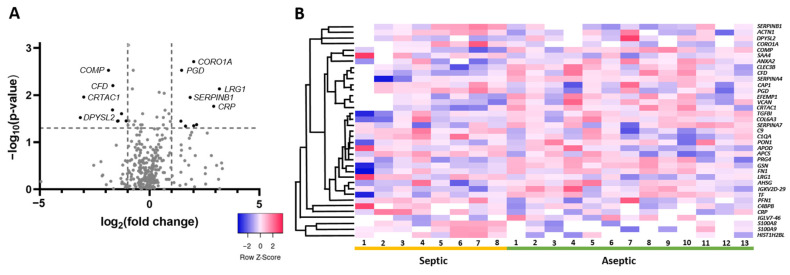
Visualization of differentially abundant proteins in the SF of patients with septic implant failure compared to aseptic failure. (**A**): The volcano plot visualizes the protein distribution based on the fold change and the *p*-value in septic SF. Dashed lines indicate the threshold values; the horizontal line marks a *p*-value < 0.05, while the vertical lines mark log_2_ FC > 1 and <−1. (**B**): The heat map shows the distribution of all 37 differentially abundant proteins in each sample and the corresponding trend of enrichment (red) vs. decrease (blue) in concentration.

A statistical comparison of the proteins that were abundant in at least 60% of the samples within one group revealed the presence of 37 proteins of varying abundance (*p* < 0.05). Of these, 17 proteins (46%) showed a higher abundance in the septic group. Considering the threshold of at least a twofold change (log_2_ ≥ 1) for higher abundance and a decrease of 0.5 (log_2_ ≤ −1), nineteen proteins were considered, leaving nine proteins with at least a twofold change in abundance and ten proteins whose abundance decreased by at least half (Table 3). The proteins with the highest fold change in the septic group included the known marker proteins c-reactive protein (7.57-fold) and the calprotectin components protein *S100-A8* (4.41-fold) and protein S100-A9 (3.1-fold). The protein with the highest fold change was leucine-rich alpha-2-glycoprotein 1 (*LRG1*) (9.07-fold) (Figure 3A, Table 2). Looking at the protein distribution in the different SF samples, the trend towards enrichment of these proteins in septic SF samples in contrast to aseptic SF samples is observable. However, patient-specific differences are also recognizable (Figure 3B).

In order to place the differentially abundant proteins into a biological context, pathway enrichment analysis was performed. Significantly enriched pathways were mainly related to hemostasis (platelet activation, signaling, and aggregation), the innate immune system (neutrophil degranulation and the complement system), extracellular matrix organization, and metabolism of proteins (Table 4). The majority of proteins that showed a fold change of more than 2 in septic SF could be assigned to the immune system, including *LRG1*, *CRP*, *SERPINB1*, *S10A08*, and *S100A9*.

### 2.3. Distribution of Biomarkers in SF

**Figure 4:**
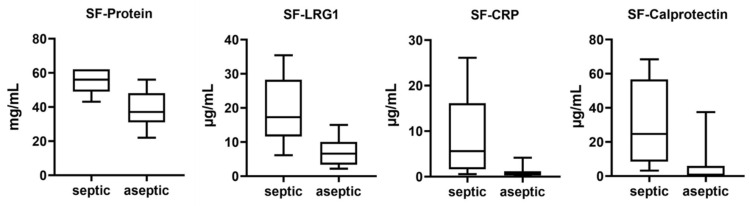
Boxplots illustrating the biomarker concentration in SF of patients with septic and aseptic implant failure.

In the SF samples, we determined the protein concentration using a modified BCA method and the concentration of the biomarkers *LRG1*, *CRP*, and calprotectin using ELISA (Table 5). With the exception of protein concentration, all values showed a large scatter (SD ≥ 50%). However, as expected from the proteomic results, in all cases, the mean protein and biomarker concentrations were significantly higher in the SF of patients with septic implant failure than in the aseptic group (*p* ≤ 0.003), and the results show a very large effect size (Cohen’s d > 1.2) (Figure 4, Table 5).

**Figure 5:**
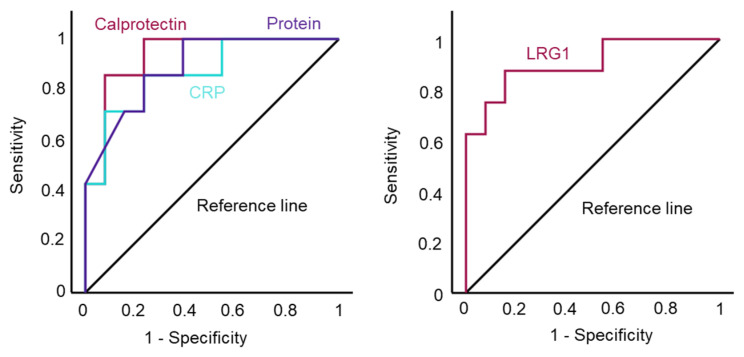
ROC (receiver operating characteristic) analysis of all parameters.

To determine a meaningful cut-off value for the diagnosis of septic implant failure using the tested biomarkers in our cohort and to evaluate the efficacy of our test, we performed a receiver operating characteristic (ROC) analysis. In all cases, ROC curves and the test accuracy determined by the area under the curve (AUC) were similar (Figure 5). With the exception of *LRG1*, cut-off values with a sensitivity of 87.5% and a specificity of around 75% were determined for all biomarkers, with *LRG1* having a higher specificity of 84.6%. The cut-off values are shown in Table 4.

## 3. Discussion

### 3.1. The SF Proteome and the Influence of Abundant Protein Depletion

In this study, we utilized the emerging discipline of proteomics to investigate the proteomic profile of synovial fluid (SF) from patients undergoing revision surgery for septic or aseptic implant failure. 

SF is an ultrafiltrate of blood plasma containing plasma proteins as the main component, but also proteins from the synovial membrane and cartilage. The protein composition in SF can reflect the pathophysiological conditions, as changes in cell metabolism and the structure of cartilage and synovial tissue in joint injuries and diseases can lead to a change in SF protein composition [23]. Therefore, it can be used for the detection of new biomarkers. In our study, we found that most proteins in SF could be assigned to the plasma proteome and that the two proteins with the highest abundance were albumin and immunoglobulin heavy constant gamma 1, analogous to blood plasma. This is in line with the study by Bennike et al., who investigated the proteome of the synovial fluid of healthy porcine joints using an LC-MS/MS-based approach [24]. The high abundance of albumin and IgGs in the synovial fluid may mask less abundant proteins that could be of interest as potential biomarkers. For proteomics, immunodepletion of these proteins is still proposed and performed in the literature [25,26,27,28,29,30]. However, depletion can result in the removal of non-targeted proteins through non-specific association with the depletion columns or with capture antibodies, which can eliminate many valuable biomarkers [31,32,33,34]. Depletion may also not improve the relative protein abundance compared to the non-depleted sample, or proteins with low abundance may still be below the detection limit [34]. In our case, albumin and IgG depletion of the SF prior to LC-MS/MS proteomic analysis with the PureProteome Albumin/IgG depletion kit (Sigma Aldrich, St. Louis, MO, USA) resulted in non-specific depletion, including depletion of CRP, and significant changes in protein abundance of 57% of all proteins.

### 3.2. The Use of Proteomics for the Detection of New Biomarkers

The concept of searching for new biomarkers for the diagnosis of PJI using a proteomic approach in which SF, sonicate fluid, or periprosthetic tissue is analyzed is not new. Since many common markers such as CRP, leukocyte esterase, and alpha-defensin are associated with an immunogenic response according to the ICM criteria for the diagnosis of PJI, the investigation of an upregulation of inflammatory proteins is obvious [13]. In our study, pathway enrichment analysis revealed that the significantly enriched pathways in septic implant failure were mainly related to hemostasis (platelet activation, signaling, and aggregation), the innate immune system (neutrophil degranulation and the complement system), extracellular matrix organization, and protein metabolism. We could show that the proteins with the highest fold change could be assigned to the immune system including LRG1, CRP, SERPINB1, S10A08, and S100A9. A study by Chen et al. found similar upregulated biological pathways when investigating the periprosthetic tissue of patients with septic and aseptic implant failure using an LC-MS/MS-based approach, including complement and coagulation cascades, phagocytosis, neutrophil activation, and other pathways. The authors found 435 differentially expressed proteins, 213 of which were upregulated in PJI [35]. However, a detailed comparison of the significantly upregulated proteins with our results was not possible because a corresponding protein list was missing. 

Jacovides et al. analyzed the SF from patients undergoing revision arthroplasty due to septic or aseptic implant failure for 46 inflammatory proteins using a multiplex ELISA protocol. In the ROC analysis, they found an AUC of over 0.7 for eighteen proteins and an AUC of over 0.9 for five proteins, including CRP [22]. Of these forty-six proteins examined, we found only ten proteins in the SF proteome, including alpha-1-antitryosin, alpha-2-macroglobulin, CRP, complement C3, haptoglobin, matrix metalloproteinase (MMP) 2, MMP3, MMP9, vitamin D-binding protein, and van Willebrand factor. The only protein that reached statistical significance was CRP, one of the proteins that had an AUC greater than 0.9 in the study described. 

Fisher et al. also investigated a 92-protein inflammation panel performed on sonicate fluid and found 37 proteins differentially expressed in PJI. Using ROC analysis, CCL20, OSM, IL6, and EN-RAGE were identified as the most promising markers for PJI [21]. Again, none of these proteins were found in the SF proteome within our study. The absence of interleukins and C-C motif chemokines may be due to the fact that the detection limit was not reached or that the sonicate fluid contains these proteins in higher concentrations or that the methods are different.

Studies examining the proteome of septic implant failure more generally were more congruent with our study. Differences in the results could be due to the sample size, which was larger in these studies, or to the material examined. In a study by Wang et al., which examined the SF proteome using mass spectrometry, lactotransferrin (LTF), proteinase 3 (PRTN3), and myeloid cell nuclear differentiation antigen (MNDA) were proposed as new biomarkers for PJI [36]. We found all three proteins in the SF proteome showing a higher abundance in SF of patients with septic implant failure. However, they were not statistically significant, and MNDA and PRTN3 were low-abundance proteins found in less than 60% of all samples within a group. Of the 17 proteins that showed higher abundance in the septic group, 11 proteins were also upregulated in the aforementioned study (CRP, S100A8/A9, CORO1A, HISTH2HBL, SERPINB1, PGD, APCS; ACTN1, APOD, CAP1). Of the proteins with a log_2_ fold change >1, all proteins except LRG1 and SAA4 are included. 

In a study by Fisher et al., in which sonicate fluid was analyzed using LC-MS/MS, 35 statistically significant proteins with a log_2_ fold change value ≥2 or ≤−2 were found [20]. Consistent with this, six proteins, including S100A8/A9, LRG1, ANXA2, PRG4, and CRTAC1, were also significantly altered in our study, with a log_2_ fold change ≥1 or ≤−1 and similar trends. Eleven more of the significantly altered proteins were found in the 60% interval. Of these proteins, six showed a tendency to be found in higher concentrations in septic SF, including LTF, LCN2, MPO, CTDG, MMP9, and PYGL. Interestingly, in contrast to their other study, which examined the inflammation panel, there is no overlap between the results of these two studies, as no interleukins and C-C motif chemokines were found in the proteome. The authors explain these results with the specific characteristics of the individual methods [20].

### 3.3. Significance of the Identified Biomarkers 

While CRP and calprotectin are already in focus as biomarkers for the diagnosis of PJI and are excellently reviewed elsewhere [17,37,38,39,40], LRG1 has never been linked in this context. Although the authors of the previous study also found that LRG1 is upregulated in septic SF, they did not attribute any significance to this [20]. In our study, it was the protein with the highest fold change, and quantification in SF led to statistically significant results showing the best ROC curve, making it an interesting new biomarker for the diagnosis of PJI. 

LRG1 is a highly conserved glycoprotein that is synthesized by hepatocytes and neutrophils under physiological conditions. It was first discovered in human serum in 1977 and has gained considerable interest in recent years, as it has been found to be involved in a variety of pathological processes, including cardiovascular diseases, diabetes, neurological diseases, inflammatory disorders, and cancer. As part of the innate immune response, the level of the acute phase protein LRG1 increases following microbial infections and other inflammatory stimuli [41]. Studies have shown elevated LRG1 levels in patients suffering from rheumatoid arthritis [42,43,44,45]. In osteoarthritis, LRG1 expression was found to be upregulated in articular cartilage and subchondral bone. Here it was shown that LRG1 contributes to angiogenesis-induced new bone formation by promoting the invasion of new blood vessels and the recruitment of MSC cells into the subchondral bone of osteoarthritic joints [46]. However, whether LRG1 is suitable for the diagnosis of PJIs still needs to be evaluated in further studies.

In our study, we found a statistically significant higher protein content in the septic group (*p* = 0.002), with 55 ± 7 mg/mL compared to 39 ± 10 mg/mL in the aseptic group, which is in line with the literature. The protein concentration in healthy joints is about 1/3 of the protein concentration in plasma and is 19–28 mg/mL [23]. However, it is known that the volume of synovial fluid, the protein concentration, and the composition change drastically in active joint diseases, e.g., to around 30 mg/mL in osteoarthritis and 40 mg/mL in artificial joints [24,47].

### 3.4. Limitations of Our Study

The main limitation of our study is the size of our patient population, which is too small to draw statistically accurate conclusions. In our study, we see very similar results for all examined biomarkers with almost the same sensitivity and specificity in ROC analysis. We are aware that the ROC calculations in our study are of low general validity, and we only used them to determine meaningful cut-off values within our small patient population. The calculation of the standard error (SE) according to [48] for our ROC analysis results in an error of 8% with a mean AUC of 0.9 and a sample size of eight patients to thirteen controls. To halve the SE, we would have to quadruple our sample size to 84 (32 diseased and 52 control subjects). In this case, however, the prevalence of the disease is not considered. Based on the data for PJI as a reason for revision surgery in the German registry, we would assume a prevalence of 16%. According to a modified Cochrane formula, 263 patients would have to be examined to be statistically valid with a confidence interval of 95%, a margin of error of 10%, a sensitivity of 87.5%, and 16% disease prevalence. If the desired degree of precision is to be halved to 5%, 1051 patients are required [49,50].

Another limitation of our study is that the gender ratio in the control group is unbalanced, as this group consists predominantly of women and gender-specific factors may influence the study results. It is known that women suffer more frequently from osteoarthritis and spinal disorders as well as certain soft tissue tumors and that the effectiveness of analgesics for treating conditions such as osteoarthritis varies [51]. Regarding arthroplasty failure, however, the influence of gender-specific factors has not been fully clarified and is sometimes described controversially in the literature [52,53,54,55,56,57].

## 4. Materials and Methods

### 4.1. Study Design and Sample Collection

The study was approved by the Ethics Committee of the Medical Faculty of the University of Duisburg-Essen, Germany (No.: 18-8042-BO) and was conducted in accordance with the Declaration of Helsinki. Between May 2020 and March 2021, 21 patients were recruited who underwent revision surgery due to persistent pain after hip, knee, or shoulder arthroplasty. The presence of a periprosthetic joint infection (PJI) was diagnosed based on the 2018 consensus definition of periprosthetic hip and knee joint infections [12]. Before inclusion in the study, all patients had given their written informed consent. Inclusion criteria were complete clinical and laboratory data and a sufficient amount of synovial fluid (SF) for all determinations and to allow the diagnosis of periprosthetic joint infection (PJI). Exclusion criteria were signs of early postoperative PJI (8 weeks) due to reduced reliability of synovial and serologic markers shortly after surgery, inflammatory comorbidities (rheumatism, chronic bowel disorder), metallosis, and previous or concomitant antibiotic therapy. SF was harvested intraoperatively via joint puncture avoiding an admixture with blood using an 18-gauge needle. The SF was transferred into sterile tubes, stored on ice, and transported to the laboratory within 30 min, where the SF samples were centrifuged at 8000× *g* for 1 min. The supernatant was collected and aliquoted under sterile conditions using a laminar flow bench and stored at −80 °C until use. Soft tissue samples from the joint space were collected intraoperatively and were microbiologically investigated (microscopy, Gram staining, 14-day in vitro culture).

### 4.2. Protein and Biomarker Quantification in the SF

Protein quantification was performed by the BCA method [58] using a modified micro-BCA assay (Thermo Fischer Scientific, Rockford, IL, USA) as previously described [59]. In brief, 50 µL of the phosphate buffered saline (DPBS, Gibco, Thermo Fischer, Bend, OR, USA) diluted protein sample was mixed with 250 µL of the BCA reagent, incubated at 60 °C for 1 h, and measured at 570 nm in a plate reader (Multiskan Ascent, Thermo-Fischer, Rockford, IL, USA). The protein concentrations were measured in triplicates at three different dilutions (1:1000, 1:2000, 1:3000). The SF concentrations of leucine-rich alpha-2-glycoprotein 1 (LRG1), c-reactive protein (CRP), and calprotectin were determined by ELISA (human LRG1, CRP and calprotectin ELISA kits, Abcam, Cambridge, UK) according to the manufacturer’s protocol and measured at 450 nm using a Multiscan Ascent Reader (Thermo Scientific). The SF was diluted in a range of 1:400–1:20,000.

### 4.3. Proteome Measurement and Preparation

Single-pot, solid-phase-enhanced sample preparation: Protein samples were first digested following the SP3 (single-pot, solid-phase-enhanced sample preparation) protocol published by Hughes et al. in 2019 [60]. The cleared tryptic digests were then desalted using home-made C18 StageTips as described by Rappsilber et al. [61]. 

Proteome analysis: LC-MS/MS experiments were performed on an Orbitrap Elite instrument (Thermo, Waltham, MA, USA) that was coupled to an EASY-nLC 1000 liquid chromatography (LC) system (Thermo). Precursor ion scanning was performed in the Orbitrap analyzer (FTMS; Fourier Transform Mass Spectrometry) in the scan range of *m*/*z* 300–1800 and at a resolution of 60,000 with the internal lock mass option turned on (lock mass was 445.120025 *m*/*z*, polysiloxane) [62].

Peptide and protein identification using MaxQuant: RAW spectra were submitted to an Andromeda search [63] in MaxQuant (2.0.3.0.) using the default settings [64]. Label-free quantification and match-between-runs was activated [65]. The MS/MS spectra data were searched against the Uniprot H. sapiens reference database (UP000005640_9606_OGPP.fasta, 20,589 entries, downloaded 10 January 2022). All searches included a contaminants database search (as implemented in MaxQuant, 245 entries). Further analysis and filtering of the results was carried out in Perseus v1.6.10.0. [66].

Bioinformatics analysis: Pathway enrichment analysis was conducted using the reactome online analysis tool [67,68]. Significantly enriched pathways were defined based on the *p*-value cut-off (false discovery rate (FDR)) below 0.05.

The full description of the proteome measurement and preparation can be found in Supplementary Document S1.

### 4.4. Statistics and Data Visualization

The statistics were compiled using the software package SPSS (IBM, Armonk, NY, USA, v.29.0.1.0). The normal distribution of the data was tested using the Kolmogorov–Smirnov test. Normally distributed continuous data were compared using student’s *t*-test and expressed as mean ± standard deviation (SD). Non-normally distributed continuous data were compared using the Mann–Whitney U test and expressed as mean values. In most cases, the proteomic data were not normally distributed. Statistical results were considered significant if the *p*-value was < 0.05.

The following criteria were used to determine the differential abundance of proteins in the septic/aseptic group: (i) proteins that were identified in at least 60% of the samples in at least one group and (ii) for which a nominally significant difference in protein abundance was observed between conditions (unadjusted *p* < 0.05). The relationship between protein abundance in both groups was analyzed by linear regression.

The discriminatory power of the biomarker was determined using the receiver operating characteristics (ROC) analysis, in which the sensitivity, specificity, area under the curve (AUC), and its 95% confidence interval (CI) were determined for each cut-off value.

Pie and bar charts were created in Microsoft Excel (MS Office Professional Plus 2016). The heatmap was created using the online tool http://heatmapper.ca/ (accessed on 5 December 2023) [69]. Volcano and boxplots were created in prism v.9.5.1. Scatter and ROC diagrams were created with SPSS (IBM, v.29.0.1.0).

## 5. Conclusions

When examining the proteome of the synovial fluid of patients undergoing revision surgery for septic or aseptic implant failure using LC-MS/MS, we found elevated levels of the known biomarkers *CRP* and calprotectin for the diagnosis of PJI. However, in contrast to other studies, we identified significantly higher levels of *LRG1*, a currently discussed new biomarker for inflammatory diseases. In the future, elevated *LRG1* levels in SF may facilitate the diagnosis of PJI, but their significance needs to be further investigated using larger samples.

## Figures and Tables

**Table 1:** Demographic and clinical baseline data. Continuous data are expressed as mean and standard deviation and categorical data as number and percentage (%).

Variable		Septic	Aseptic	*p*-Value
Number of patients		8 (38%)	13 (62%)	
Female:Male		4:4 (50%)	11:2 (85%)	0.210
Age [years]		68.5 ± 13.3	67.8 ± 11.0	0.893
BMI		29.4 ± 5.3	31.6 ± 5.6	0.742
Revision site	hip	6 (75%)	7 (54%)	
	knee	-	6 (46%)	
	shoulder	2 (25%)	-	
Comorbidities				
Hypertension		4 (50%)	4 (31%)	
Obesity		6 (75%)	5 (38%)	
Osteoporosis		1 (12%)	1 (8%)	
Heart disease		2 (25%)	3 (23%)	
Kidney disease		1 (13%)	-	
Parkinson’s disease		1 (13%)	-	
Diabetes		3 (38%)	-	
Hypothyroidism		-	1 (8%)	
COPD		-	1 (8%)	

**Table 2:** Preoperative serum parameters of patients within one group as mean and standard deviation (for normally distributed parameters) and *p*-values. Abbreviations: CRP—c reactive protein; PTT—partial thromboplastin time; *—statistically significant.

Plasma/Blood Parameter	Septic	Aseptic	*p*-Value	Normal Values
Leukozytes [1 per nL]	8.6 ± 1.9	7.7 ± 2.8	0.410	4.5–12.7
Thrombocytes [1 per nL]	295.9 ± 111.3	275.6 ± 49.1	0.570	173.0–390.0
Sodium [mmol/L]	138.8 ± 3.5	140.6 ±2.3	0.157	136.0–145.0
Potassium [mmol/L]	4.4 ± 0.4	4.8 ± 0.4	0.056	3.5–5.1
Hemoglobin [g/dL]	12.3 ± 2.5	13.7 ± 1.2	0.170	11.9–14.6
Creatinine [mg/dL]	1.0	0.9	0.514	0.5–0.9
CRP [mg/dL]	5.1	0.5	0.002 *	<0.5
PTT [s]	30.7	28.6	0.218	23.9–33.2
Quick [%]	91.8 ± 23.5	97.6 ± 10.2	0.436	74–120

**Table 3:** List of proteins with statistically significant changes (*p* < 0.05) in abundance in the SF of patients with septic implant failure compared to aseptic failure. A fold change (FC) greater than 1 means a higher abundance in the SF of septic patients, while an FC less than 1 means lower abundance. Proteins above the first dashed line are enriched in septic SF and show at least a twofold increase in abundance compared to proteins in aseptic implant failure (log_2_ FC > 1). Proteins below the second dashed line show a reduction in abundance by at least half in septic SF (log_2_ FC < 1).

Protein	Gene Name	FC	log_2_ FC	*p*-Value
Leucine-rich alpha-2-glycoprotein	*LRG1*	9.07	3.18	0.007
C-reactive protein	*CRP*	7.57	2.92	0.017
Protein S100-A8	*S100A8*	4.42	2.14	0.042
Coronin-1A	*CORO1A*	4.03	2.01	0.002
Histone H2B type 1-L	*HIST1H2BL*	4.02	2.01	0.045
Leukocyte elastase inhibitor	*SERPINB1*	3.60	1.85	0.011
Protein S100-A9	*S100A9*	3.11	1.63	0.046
6-phosphogluconate dehydrogenase	*PGD*	2.75	1.46	0.003
Serum amyloid A-4 protein	*SAA4*	2.69	1.43	0.036
C4b-binding protein beta chain	*C4BPB*	1.92	0.94	0.036
Serum amyloid P-component	*APCS*	1.84	0.88	0.025
Alpha-actinin-1	*ACTN1*	1.63	0.70	0.021
Complement component C9	*C9*	1.59	0.67	0.009
Complement C1q subcomponent subunit A	*C1QA*	1.54	0.62	0.030
Apolipoprotein D	*APOD*	1.54	0.62	0.043
Profilin-1	*PFN1*	1.35	0.44	0.009
Adenylyl cyclase-associated protein 1	*CAP1*	1.26	0.33	0.029
Collagen alpha-3(VI) chain	*COL6A3*	0.80	−0.32	0.029
Serum paraoxonase/arylesterase 1	*PON1*	0.79	−0.34	0.043
Alpha-2-HS-glycoprotein	*AHSG*	0.79	−0.35	0.043
Thyroxine-binding globulin	*SERPINA7*	0.75	−0.41	0.043
Transforming growth factor-β-induced	*TGFBI*	0.63	−0.67	0.036
Immunoglobulin kappa variable 2D-29	*IGKV2D-29*	0.60	−0.73	0.043
Kallistatin	*SERPINA4*	0.60	−0.74	0.003
Serotransferrin	*TF*	0.58	−0.79	0.007
EGF-containing fibulin extracellular matrix p. 1	*EFEMP1*	0.52	−0.94	0.035
Gelsolin	*GSN*	0.51	−0.96	0.001
Tetranectin	*CLEC3B*	0.50	−0.99	0.043
Annexin A2	*ANXA2*	0.48	−1.07	0.035
Fibronectin	*FN1*	0.41	−1.28	0.025
Versican core protein	*VCAN*	0.37	−1.43	0.035
Proteoglycan 4	*PRG4*	0.36	−1.46	0.036
Complement factor D	*CFD*	0.31	−1.68	0.006
Immunoglobulin lambda variable 7-46	*IGLV7-46*	0.31	−1.69	0.021
Cartilage oligomeric matrix protein	*COMP*	0.27	−1.87	0.003
Cartilage acidic protein 1	*CRTAC1*	0.12	−3.00	0.011
Dihydropyrimidinase-related protein 2	*DPYSL2*	0.11	−3.16	0.030

**Table 4:** Abridged compilation of the significantly enriched pathways from the analysis of the differentially abundant proteins between septic and aseptic SF. Pathways belonging to the same “parental pathway” were grouped based on the position of the pathway in the hierarchy. Proteins associated with these pathways and the complete list are presented in Supplementary Table S2. Abbreviation: FDR—false discovery rate.

Main Parental Pathway	Enriched Pathway	Total Proteins in the Pathway	Assigned Proteins	FDR
Hemostasis	Platelet activation, signaling, and aggregation	293	9	1.47 × 10^−5^
Immune System	Innate Immune System	1341	15	1.79 × 10^−4^
	Neutrophil degranulation	478	9	3.47 × 10^−4^
	Complement Cascade	156	6	3.1 × 10^−4^
Extracellular matrix organization	ECM proteoglycans	79	4	3.25 × 10^−3^
	Integrin cell surface interactions	86	3	3.41 × 10^−2^
Metabolism of proteins	Amyloid fiber formation	89	4	4.59 × 10^−3^
	Post-translational protein phosphorylation	109	4	7.93 × 10^−3^
	Regulation of Insulin-like Growth Factor (IGF) transport	127	4	1.11 × 10^−2^

**Table 5:** Diagnostic accuracy in the diagnosis of septic implant failure using SF biomarkers. AUC: area under the curve, *LRG1*: leucine-rich glycoprotein 1, *CRP*: c-reactive protein, *—statistically significant.

SF Parameter	Septic	Aseptic	Cut-Off	Sensitivity [%]	Specificity [%]	AUC	*p*-Value	Cohen’s d
Protein [mg/mL]	55.0 ± 5.7	38.8 ± 10.3	47	87.5	76.9	0.89	0.002 *	1.72
* LRG1 * [mg/mL]	19.4 ± 9.4	7.1 ± 4.1	10.95	87.5	84.6	0.90	0.001 *	1.88
* CRP * [µg/mL]	9.1 ± 8.6	0.9 ± 1.1	1.1	87.5	76.9	0.87	0.003 *	1.56
Calprotectin [µg/mL]	31.9 ± 23.8	5.2 ± 10.7	4.63	87.5	76.9	0.93	0.001 *	1.59

## Data Availability

The mass spectrometry proteomics data for the on-bead digestions have been deposited to the ProteomeXchange Consortium via the PRIDE partner repository (https://www.ebi.ac.uk/pride/archive/) with the dataset identifier PXD047507.

## Supplementary Materials

The following supporting information can be downloaded at: https://www.mdpi.com/article/10.3390/antibiotics13040346/s1, Supplementary Table S1: List of proteins identified with at least 2 peptides, Supplementary Table S2: List of Proteins in at least 60% of a group, Supplementary Table S3: Significantly enriched pathways. Supplementary Document S1: Full description of the proteome method.
