# Supplementary material for: Differences in the Synovial Fluid Proteome of Septic and Aseptic Implant Failure
Source: Antibiotics (Basel). 2024 Apr 9;13(4):346. doi: 10.3390/antibiotics13040346 (PMC11047638; doi:10.3390/antibiotics13040346)
Supplement: Supplementary file 1 [file antibiotics-13-00346-s001.zip › Supplementary Document S1.pdf]

Full description of proteome measurement and preparation

Single-pot, solid-phase-enhanced sample preparation: Protein samples were first digested following the SP3 (single-pot, solid-phase-enhanced sample preparation) protocol published by Hughes et al. in 2019 [1]. A volume equivalent to 15 µg of protein from each sample was taken up in 100 µL 1× SP3 lysis buffer (final concentrations: 5% sodium dodecyl sulfate (SDS); 200 µL 40 mM chloroacetamide; 10 mM tris(2-carboxyethyl)phosphine hydrochloride (TCEP); 200 mM 2-(4-(2-hydroxyethyl)-1-piperazinyl)-ethanesulfonic acid (HEPES) pH 8; 200 µL 40 mM chloroacetamide) and heated for 5 min at 90°C. After cooling the samples to room temperature, a mix of pre-equilibrated hydrophobic and hydrophilic SeraMag speed beads (75 µg of each; Cytiva) was added to the denatured and alkylated protein solution (bead-to-protein ratio 10:1) and gently mixed. One volume of 100% ethanol (EtOH) was added and the protein/bead/EtOH suspension was incubated for 20 min at 24°C on a Thermomixer C (Eppendorf) while shaking at 1200 rpm. After this binding step, the beads were collected on a magnet (minimum of 4 min) and washed 4× with 80% EtOH. After the final wash step, the beads were taken up in 25 mM ammonium bicarbonate (ABC) with 1 µg trypsin (protein-to-trypsin ratio 30:1), sonicated for 5 minutes at 37°C, then transferred to a Thermomixer C and incubated overnight at 37°C with shaking at 1300 rpm. Afterwards, the samples were acidified with formic acid (FA, final concentration 1%), the beads were collected, and the supernatant was transferred to a fresh Eppendorf tube. The cleared tryptic digests were then desalted using home-made C18 StageTips as described by Rappsilber et al. [2]. Briefly, peptides were immobilized and washed on a 2-disc C18 StageTip. After elution from the StageTips, samples were dried using a vacuum concentrator (Eppendorf) and the peptides were taken up in 0.1% formic acid solution (15 µL) and directly used for LC-MS/MS experiments.

Proteome analysis: LC-MS/MS experiments were performed on an Orbitrap Elite instrument (Thermo) that was coupled to an EASY-nLC 1000 liquid chromatography (LC) system (Thermo). The LC was operated in the one-column mode. The analytical column was a fused silica capillary (75 µm × 42 cm) with an integrated fritted emitter (15 µm; CoAnn Technologies) packed in-house with Kinetex C18-XB core shell 1.7 µm resin (Phenomenex). The analytical column was encased by a column oven (Sonation) and attached to a nanospray flex ion source (Thermo). The column oven temperature was adjusted to 50 °C during data acquisition. The LC was equipped with two mobile phases: solvent A (0.2% formic acid, FA, in water) and solvent B (0.2% FA, 19.8% water and 80% acetonitrile, ACN). All solvents were of UPLC grade (Honeywell). Peptides were directly loaded onto the analytical column with a maximum flow rate that would not exceed the set pressure limit of 980 bar (usually around 0.6 – 1.0 µL/min). Peptides were subsequently separated on the analytical column by running a 140 min gradient of solvent A and solvent B (start with 3% B; gradient 3% to 7% B for 5 min; gradient 7% to 20% B for 90 min; gradient 20% to 46% B for 30 min; gradient 46% to 100% B for 4 min and 100% B for 11 min) at a flow rate of 350 nl/min. The mass spectrometer was operated using Xcalibur software (version v3.0.63 SP1.48). The mass spectrometer was set in the positive ion mode. Precursor ion scanning was performed in the Orbitrap analyzer (FTMS; Fourier Transform Mass Spectrometry) in the scan range of m/z 300-1800 and at a resolution of 60000 with the internal lock mass option turned on (lock mass was 445.120025 m/z, polysiloxane) [3]. Product ion spectra were recorded in a data-dependent fashion in the ion trap (ITMS) in a variable scan range and at a rapid scan rate. The ionization potential (spray voltage) was set to 1.8 kV. Peptides were analyzed using a repeating cycle consisting of a full precursor ion scan (3.0 × 106 ions or 50 ms) followed by 15 product ion scans (1.0 × 104 ions or 60 ms) where peptides are isolated based on their intensity in the full survey scan (threshold of 500 counts) for tandem mass spectrum (MS2) generation that permits peptide sequencing and identification. Collision-induced dissociation (CID) energy was set to 35% for the generation of MS2 spectra. During MS2 data acquisition, dynamic ion exclusion was set to 30 seconds with a maximum list of excluded ions consisting of 500 members and a repeat count of one. Ion injection time prediction, preview mode for the FTMS, monoisotopic precursor selection, and charge state screening were enabled. Only charge states higher than 1 were considered for fragmentation.

Peptide and protein identification using MaxQuant: RAW spectra were submitted to an Andromeda search [4] in MaxQuant (2.0.3.0.) using the default settings [5]. Label-free quantification and match-between-runs was activated [6]. The MS/MS spectra data were searched against the Uniprot H. sapiens reference database (UP000005640_9606_OGPP.fasta, 20589 entries, downloaded 10/1/2022). All searches included a contaminant database search (as implemented in MaxQuant, 245 entries). The contaminants database contains known MS contaminants and was included to estimate the level of contamination. Andromeda searches allowed oxidation of methionine residues (16 Da) and acetylation of the protein N-terminus (42 Da) as dynamic modifications and the static modification of cysteine (57 Da, alkylation with iodoacetamide). Enzyme specificity was set to "Trypsin/P" with two missed cleavages allowed. The instrument type in Andromeda searches was set to Orbitrap and the precursor mass tolerance was set to ±20 ppm (first search) and ±4.5 ppm (main search). The MS/MS match tolerance was set to ±0.5 Da. The peptide spectrum match FDR and the protein FDR were set to 0.01 (based on target-decoy approach). Minimum peptide length was 7 amino acids. For protein quantification, unique and razor peptides were allowed. Modified peptides were allowed for quantification. The minimum score for modified peptides was 40. Label-free protein quantification was switched on, and unique and razor peptides were considered for quantification with a minimum ratio count of 2. Retention times were recalibrated based on the built-in nonlinear time-rescaling algorithm. MS/MS identifications were transferred between LC-MS/MS runs with the "match between runs" option, in which the maximal match time window was set to 0.7 min and the alignment time window set to 20 min. The quantification is based on the "value at maximum" of the extracted ion current. At least two quantitation events were required for a quantifiable protein. Further analysis and filtering of the results was carried out in Perseus v1.6.10.0. [7]. Comparison of protein group quantities (relative quantification) between different MS runs is based solely on the LFQs as calculated by MaxQuant, MaxLFQ algorithm [6].

Bioinformatics analysis: Pathway enrichment analysis was conducted using the reactome online analysis tool [8,9]. Significantly enriched pathways were defined based on the p-value cut-off (false discovery rate (FDR)) below 0.05.

Literature

1. Hughes, C.S.; Moggridge, S.; Müller, T.; Sorensen, P.H.; Morin, G.B.; Krijgsveld, J. Single-pot, solid-phase-enhanced sample preparation for proteomics experiments. *Nat. Protoc.* **2019**, *14*, 68–85, doi:10.1038/s41596-018-0082-x.
2. Rappsilber, J.; Mann, M.; Ishihama, Y. Protocol for micro-purification, enrichment, pre-fractionation and storage of peptides for proteomics using StageTips. *Nat. Protoc.* **2007**, *2*, 1896–1906, doi:10.1038/nprot.2007.261.
3. Olsen, J.V.; Godoy, L.M.F. de; Li, G.; Macek, B.; Mortensen, P.; Pesch, R.; Makarov, A.; Lange, O.; Horning, S.; Mann, M. Parts per million mass accuracy on an Orbitrap mass spectrometer via lock mass injection into a C-trap. Mol. Cell. *Proteomics* **2005**, *4*, 2010–2021, doi:10.1074/mcp.T500030-MCP200.
4. Cox, J.; Neuhauser, N.; Michalski, A.; Scheltema, R.A.; Olsen, J.V.; Mann, M. Andromeda: a peptide search engine integrated into the MaxQuant environment. *J. Proteome Res.* **2011**, *10*, 1794–1805, doi:10.1021/pr101065j.
5. Cox, J.; Mann, M. MaxQuant enables high peptide identification rates, individualized p.p.b.-range mass accuracies and proteome-wide protein quantification. *Nat. Biotechnol.* **2008**, *26*, 1367–1372, doi:10.1038/nbt.1511.
6. Cox, J.; Hein, M.Y.; Luber, C.A.; Paron, I.; Nagaraj, N.; Mann, M. Accurate proteome-wide label-free quantification by delayed normalization and maximal peptide ratio extraction, termed MaxLFQ. Mol. Cell. *Proteomics* **2014**, *13*, 2513–2526, doi:10.1074/mcp.M113.031591.
7. Tyanova, S.; Temu, T.; Sinitcyn, P.; Carlson, A.; Hein, M.Y.; Geiger, T.; Mann, M.; Cox, J. The Perseus computational platform for comprehensive analysis of (prote)omics data. *Nat. Methods* **2016**, *13*, 731–740, doi:10.1038/nmeth.3901.
8. Fabregat, A.; Sidiropoulos, K.; Viteri, G.; Forner, O.; Marin-Garcia, P.; Arnau, V.; D'Eustachio, P.; Stein, L.; Hermjakob, H. Reactome pathway analysis: a high-performance in-memory approach. *BMC Bioinformatics* **2017**, *18*, 142, doi:10.1186/s12859-017-1559-2.
9. Jassal, B.; Matthews, L.; Viteri, G.; Gong, C.; Lorente, P.; Fabregat, A.; Sidiropoulos, K.; Cook, J.; Gillespie, M.; Haw, R.; et al. The reactome pathway knowledgebase. *Nucleic Acids Res.* **2020**, *48*, D498-D503, doi:10.1093/nar/gkz1031.
